# Medical Device Based on a Virtual Reality–Based Upper Limb Rehabilitation Software: Usability Evaluation Through Cognitive Walkthrough

**DOI:** 10.2196/68149

**Published:** 2025-04-01

**Authors:** Seojin Hong, Hyun Choi, Hyosun Kweon

**Affiliations:** 1 Department of Clinical Research Rehabilitation, National Rehabilitation Center, 58 Samgaksan-ro, Gangbuk-gu, Seoul, 01022, Republic of Korea, 82 02-901-1904; 2 Department of Healthcare and Public Health, National Rehabilitation Center, Seoul, Republic of Korea

**Keywords:** usability, cognitive walkthrough, virtual reality-based upper limb rehabilitation software, upper limb, limb rehabilitation, rehabilitation, therapist, virtual reality, VR, medical device, formative evaluation, quantitative, qualitative, occupational therapy, user safety, usability testing, software, risk factor

## Abstract

**Background:**

The use of virtual reality (VR) technology in rehabilitation therapy has been growing, leading to the development of VR-based upper-limb rehabilitation softwares. To ensure the effective use of such software, usability evaluations are critical to enhance user satisfaction and identify potential usability issues.

**Objective:**

This study aims to evaluate the usability of a VR-based upper-limb rehabilitation software from the perspective of occupational therapists. Specifically, the study seeks to identify usability challenges and provide insights to improve user satisfaction.

**Methods:**

The VR-based upper-limb rehabilitation software was tailored for therapists to operate while delivering therapy to patients. Usability testing was conducted with occupational therapists from the Korean National Rehabilitation Center using cognitive walkthroughs and surveys. Participants performed tasks that simulated real clinical scenarios, including turning the device on, assisting patients with wearing the device, and shutting it down. Observers recorded user reactions during task performance, and participants completed surveys to assess the ease of use of the user interface. This mixed-methods approach provided qualitative insights into user difficulties and their root causes.

**Results:**

Usability evaluations were conducted with 6 participants. Cognitive walkthroughs revealed potential areas for improvement in the software, including (1) enhancements to the graphical user interface for ease of use, (2) refinements in the natural user interface, and (3) better user manuals for clearer product instructions. The ease-of-use score for the user interface averaged 1.58 on a 5-point scale (1=very easy to 5=very difficult).

**Conclusions:**

This study provides valuable insights into improving user satisfaction by focusing on the needs of occupational therapists who operate a VR-based rehabilitation software. Future research should explore software refinement and clinical efficacy to maximize the therapeutic potential of such technologies.

## Introduction

### Background

Upper limb rehabilitation plays a critical role in restoring functional abilities in individuals with stroke or neurological injuries, enhancing their independence and autonomy [1,2]. Virtual reality (VR) technology has emerged as a promising tool in rehabilitation therapy, offering immersive and interactive features that can improve patient engagement and therapeutic outcomes [3-5]. Through tailored feedback and adjustable difficulty levels, VR-based rehabilitation programs can address diverse patient needs and therapeutic goals [6]. To achieve optimal clinical use, VR-based rehabilitation software must be designed to meet the needs of end users, including health care professionals such as occupational therapists, within their specific work environments. Usability evaluation is a critical step in this process, as it helps identify design flaws, reduce user errors, and enhance operational efficiency and satisfaction [7]. While existing studies on VR-based rehabilitation have largely focused on technical development and clinical efficacy [4], research on usability issues experienced by health care professionals during actual software use remains limited [3] . Specifically, there is a lack of comprehensive analysis regarding the intuitive interaction, learning curve, and practical applicability of such software in real-world clinical settings [7].

Systematic usability evaluation targeting occupational therapists, as key stakeholders in VR-based rehabilitation, is essential to identify and address usability issues. Formative evaluation methods, such as cognitive walkthroughs, provide valuable insights during the early stages of software development, enabling iterative improvements and better user experience [3].

### Usability Evaluation

Errors encountered during the use of medical devices are often linked to poorly designed interfaces rather than user mistakes. Although not all user errors result from design flaws, usability evaluations conducted before commercialization can effectively mitigate design-related issues [8,9]. Usability engineering processes play a vital role in identifying and addressing predictable user errors in clinical environments, ensuring the safety and efficiency of medical devices [10,11].

Usability evaluations can be categorized as formative or summative, depending on the timing and purpose of the evaluation [12]. Formative evaluations aim to identify usability issues and guide design improvements during the product development phase, while summative evaluations focus on validating outcomes at the final stages of development through objective data analysis [13]. Formative evaluations commonly use methods such as cognitive walkthroughs, task analysis, usability testing, and heuristic evaluation to explore and address usability challenges in depth [14].

Cognitive walkthroughs, in particular, are highly effective for evaluating software usability. They focus on assessing the system from the perspective of new users, enabling the identification of potential issues such as inconsistent interface behavior or a lack of clarity [15]. By providing insights into users’ cognitive processes, cognitive walkthroughs empower developers to design more user-friendly, efficient, and satisfying interfaces.

### Objective

This study aims to conduct a formative evaluation of a prototype VR-based upper limb rehabilitation software, focusing on its usability for occupational therapists. By identifying usability challenges and gathering feedback from intended users, this research seeks to provide actionable insights to guide iterative improvements in the software design. Ultimately, this study aims to contribute foundational data for user-centered design and optimize the software for effective clinical implementation.

## Methods

### Study Design

This study conducted a usability evaluation of a prototype VR-based upper limb rehabilitation software targeted for use by occupational therapists. The evaluation used cognitive walkthroughs and surveys, covering the entire user workflow, including activating the device, setting it up, and completing tasks. The software was assessed as an embedded system, including its hardware components.

### Formative Evaluation Procedures

The formative evaluation was conducted in the clinical rehabilitation testbed of the Korean National Rehabilitation Center. Each session lasted approximately 90 minutes per participant (Table 1). Before the evaluation, the facilitator introduced the usability testing process, including its objectives, methods, and relevant product information.

**Table 1.** Formative evaluation procedures. The formative evaluation involved practitioners and evaluators, and was conducted for 90 minutes under the guidance of a facilitator.

Composition	Details	Time (minutes)
Orientation	The facilitator explains the target product, purpose, overview, methodology, and other relevant aspects of the usability evaluation to the participant. The facilitator introduces the concept of usability evaluation to participants who are unfamiliar with it.	10
Guidance on consent	The facilitator provides the participant with detailed instructions regarding recording and transcription procedures. The facilitator ensures that the participant fully comprehends the evaluation content and voluntarily provides written consent to participate in the usability evaluation.	10
Perform cognitive walkthrough	The facilitator conducts a cognitive walkthrough following a predefined script and product usage instructions. The participant operates the medical device in accordance with the facilitator’s guidance. The observer documents and analyzes the participant’s responses during task performance.	50
Survey	The facilitator administers a survey to the participant to assess the usability of the user interface.	20

Participants were asked about their previous experience with similar devices. Subsequently, they followed a structured script guiding them through a series of tasks using the software. Participants operated the software as instructed, while observers recorded their behaviors and responses throughout the process. Post-testing, participants were asked to evaluate their satisfaction with the system based on the VR-based upper limb rehabilitation software.

**Figure 1.**
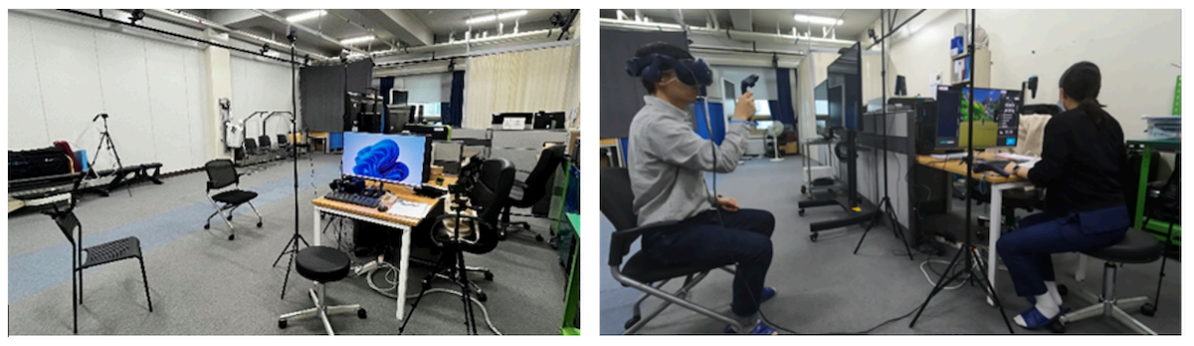
Test environment (left) and scene (right). The simulation environment is configured to resemble a rehabilitation treatment room where evaluators are used.

The usability testing was conducted in a simulated clinical environment resembling the intended use setting for the device (Figure 1). Environmental conditions, including lighting, temperature, and noise, were measured before the evaluation: lighting at 550 (SD 100) lx, temperature at 24°C (SD 2°C), relative humidity at 60% (SD 10%), and noise level at 50 (SD 5) dBA.

### Cognitive Walkthrough

The cognitive walkthrough method evaluates how well users can navigate an interface without previous training or information about the device [16]. In this study, 6 occupational therapists conducted the cognitive walkthrough, assessing the ease of navigation for specific scenarios outlined in Table 2. Observers recorded and analyzed participants’ reactions based on predefined observation sheets. Observation sheets are designed to record problems discovered during each task, user reactions and actions, and system feedback [17,18].

**Table 2.** Use scenarios for cognitive walkthrough. The facilitator verbally provided detailed subtask instructions to the occupational therapist, who performed the tasks in accordance with the usage scenario, while the simulated patient participated in the process under the occupational therapist’s guidance.

Task	Subtask
1. Reviewing the user manual	1.1. Reviewing the user manual.
2. Checking components and power supply	2.1. Checking the HMD^a^, controller, disposable face mask, PC set, and software in the PC. 2.2. Checking the product’s power supply.
3. Launching the program	3.1. Launching the “RehabwareVR” (Tech Village) icon and entering the password.
4. Creating patient information	4.1. Entering patient information. 4.2. Providing instructions on patient data consent.
5. Donning the equipment	5.1. Instructing the patient to wear the disposable face mask, HMD, and controller.
6. Running the upper rehabilitation content	6.1. Selecting one of the upper limb rehabilitation exercises, explaining it to the patient, and running.
7. Running the content	7.1. Selecting the patient and configuring the catch ball activity. 7.2. Saving these settings as User Setting 3, then starting the catch ball activity. 7.3. After completing at least five repetitions, stopping the activity, modifying the environment settings, and rerunning the activity. 7.4. Switching to the fruit transfer activity and running it. 7.5. After completing the fruit transfer activity, proceeding with the bubble activity.
8. Updating patient information	8.1. Updating the patient information.
9. Running contents after updating patient information	9.1. Selecting the patient and starting the hammering activity. 9.2. After completing the hammering activity, switching to the meteor avoidance activity. 9.3. Ending the session.
10. Doffing the product	10.1. Removing the HMD, controllers, and disposable face mask from the patient.
11. Reviewing statistical analysis results	11.1. Reviewing the data graph of the patient. 11.2. Reviewing the fruit transfer activity data for patient. 11.3 Deleting the hammering activity data for patient.
12. Ending the program	12.1. Ending the program.

a HMD: head-mounted display.

### Survey

Before the cognitive walkthrough, participants provided demographic information, clinical experience, and previous experience with similar devices. After completing the cognitive walkthrough, participants completed a survey to further assess the usability of the user interface based on the scenarios. The survey consisted of 23 items rated on a 5-point Likert scale (1=very easy to 5=very difficult). Results were analyzed using means and SDs.

### VR-Based Upper Limb Rehabilitation Software

**Figure 2.**
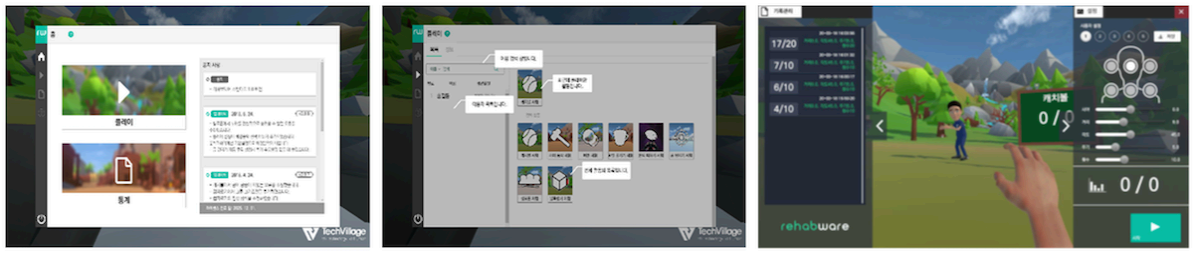
User interface used by occupational therapists in the virtual reality (VR)–based upper limb rehabilitation software: home screen (left), activity selection screen (middle), and activity options manipulation screen (right).

**Figure 3.**
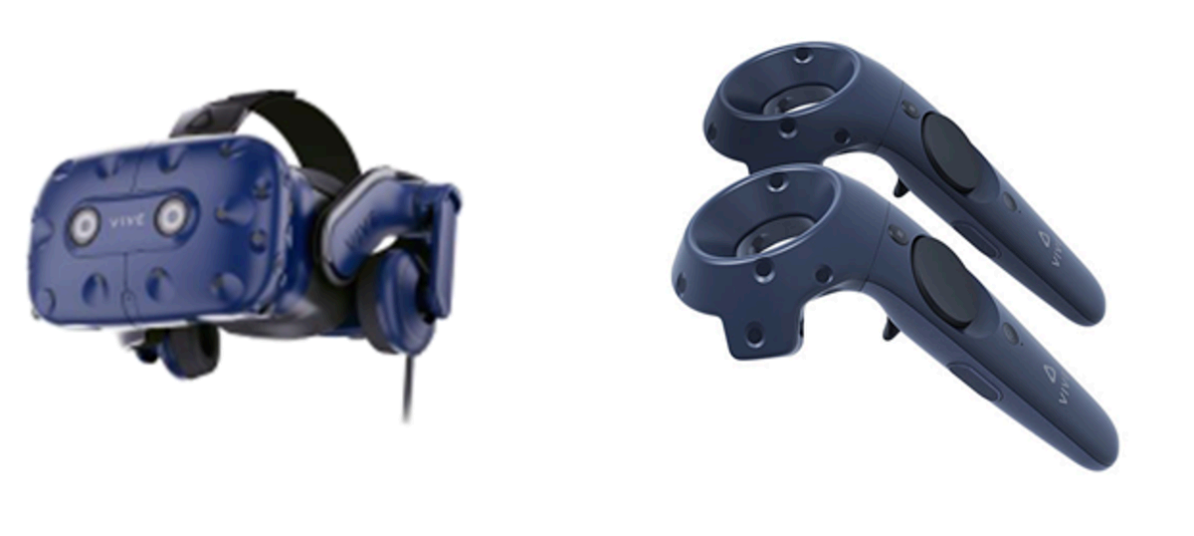
Hardware device of the virtual reality (VR)–based upper limb rehabilitation software. To use VR content, the patient wears the head-mounted device (HMD; left) on their head and holds the controller (right).

The prototype VR-based upper limb rehabilitation software used in this study was designed to improve upper limb function, including shoulder mobility, in patients with stroke or neurological injuries (Figure 2). Patients wear a head-mounted display (HMD; HTC VIVE Pro Full-Kit including VIBE base stations and controllers) and use handheld controllers to perform voluntary movements in a VR environment for rehabilitation (Figure 3). Occupational therapists, as intended users, set up the hardware, operate the software, and assist patients in improving their upper limb functions.

**Figure 4.**
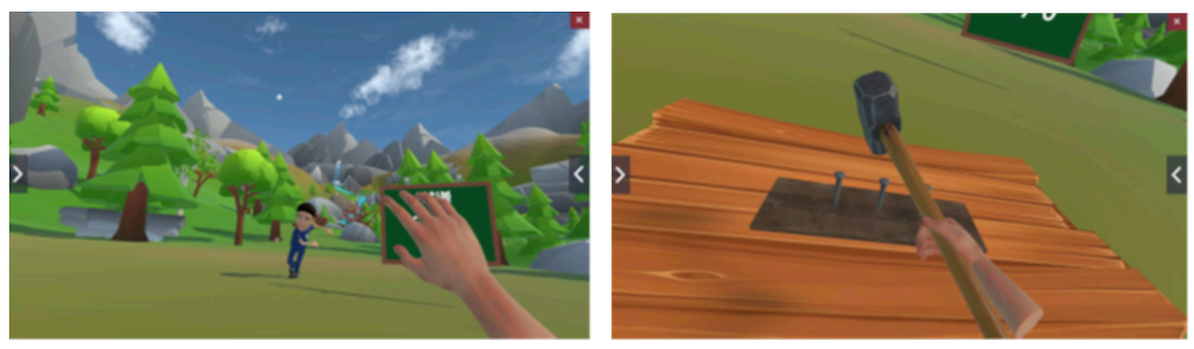
Examples of virtual reality within the patient-worn head-mounted device (HMD), such as throwing a catching balls (left) and hammering nails (right).

The software includes eight rehabilitation activities (Figure 4): (1) throwing a catching balls, (2) hammering nails, (3) popping bubbles, (4) moving fruits, (5) dodging meteors, (6) throwing balls, (7) playing the xylophone, and (8) stacking blocks.

The minimum computer requirements to run the software are Microsoft Windows 7 SP, Windows 8.1, or Windows 10, with a display resolution of 1280×72 0r higher. Recommended specifications include Microsoft Windows 10 Professional with the same resolution. The hardware used was the VIVE Pro Full-Kit.

### Participants

The usability evaluation involved 3 usability practitioners (a facilitator and 2 observers), 6 occupational therapists as participants, and 3 mock patients. The facilitator conducted the evaluation, while observers recorded and analyzed participants’ responses. The sample size of 6 participants was determined to balance cost and effectiveness, as this number is sufficient to identify most usability issues [19]. Mock patients were recruited to simulate the role of patients during testing, prioritizing safety by selecting healthy individuals. In the early stages of development, formative evaluations typically involve the use of simulated patients before including real patients. This approach ensures safety and control while being effective in identifying functional issues in devices [20,21]. Furthermore, unlike real patients, simulated patients are trained to provide consistent conditions and responses, thereby maintaining the reliability and consistency of the evaluation process [22,23]. Accordingly, this study employed simulated patients instead of real patients to conduct the evaluation. All participants were informed of the study’s purpose, methods, and procedures before testing and provided signed consent.

### Recruitment Process

Recruitment was conducted through posted announcements at the Korean National Rehabilitation Center. The inclusion criteria encompassed possession of an occupational therapist license and previous experience with rehabilitation medicine devices, such as computerized cognitive rehabilitation programs, whereas the exclusion criteria comprised individuals who did not provide consent to participate in the study. Through eligibility screening, 6 occupational therapists were selected. The mock patients were 3 healthy adults aged 19 years and older and had no experience using the evaluation product. They were provided detailed explanations of the study and coordinated testing schedules. Written informed consent was obtained from all participants.

### Ethical Considerations

This study was approved by the Institutional Review Board of the Korean National Rehabilitation Center (NRC-2023-06-041). All participants provided informed consent, and no financial compensation was offered. Data were anonymized to ensure participant privacy and confidentiality.

## Results

### Participant Characteristics

The clinical experience of the occupational therapists ranged from 6 months to 6 years, with an average of 3 years and 9 months. In addition, they had previous experience using similar medical devices, such as Comcog and RAPAEL (Neofect) (computerized cognitive rehabilitation programs), with their experience ranging from a minimum of 2 months to a maximum of 5 years (Table 3).

**Table 3.** General characteristics of participants. The participant in the cognitive walkthrough was an occupational therapist who had experience using similar medical devices.

Participant Number	Sex	Age (years)	Experience	Occupation	Experience using similar medical devices	Name of similar medical device
1	Female	28	6 years	Occupational therapist	6 months, twice a week	Comcog (Neofect), RAPAEL Smart Glove (Neofect), and RAPAEL Smart pegboard
2	Female	24	6 months	Occupational therapist	2 months, twice a week	RAPAEL
3	Female	27	5 years	Occupational therapist	5 years, twice a week	Comcog, RAPAEL, and RehaCom (Hasomed)
4	Female	25	3 years	Occupational therapist	3 years, once a week	RAPAEL and Xbox (Microsoft)
5	Male	27	3 years, 9 months	Occupational therapist	2 years, once a day	Comcog
6	Female	29	5 years, 8 months	Occupational therapist	1 year, 3 times a month	Comcog and RehaCom

### Cognitive Walkthrough Results

A total of 12 usability errors were identified using cognitive walkthrough. In task 4, “Creating Patient Information,” 4 errors were identified. In subtask 4.1, “Locating the Add Patient Icon,” participants were confused as they could not find the icon leading to the screen for adding patient information. In subtask 4.2, “Registering Patient Information,” participants hesitated when they had to enter such details as the diagnosis and dominant hand manually because moving to the next input field required a click on the mouse, as the tab key shortcut did not work. In subtask 4.3, “Guiding Patients’ Consent for Using Personal Information,” participants skipped the data consent step and almost automatically pressed the confirm button without explaining the process of consent for using personal information to the mock patient.

In task 7, “Performing the Activity,” 5 usability issues were identified. In subtask 7.1, “Selecting Activity,” participants attempted to select activities without first selecting the patient, which triggered a popup message indicating “No patient selected.” In subtask 7.2, “Configuring the Activity Settings,” participants committed multiple errors, including failure to estimate the perceived distance between the hand and the object within the virtual reality, and identifying which hand was being used for interaction. Furthermore, although the users clicked on the “User Settings” button, they failed to save the configurations. There were also challenges in adjusting precise decimal values using mouse clicks and dragging, and this time-consuming process delayed the therapeutic session. In subtask 7.4, “Changing Activities,” participants switched activities without saving current data, which led to the loss of unsaved records. In addition, some therapists had difficulty adjusting the settings after completing an activity, as they were unsure whether they needed to press the confirm button before proceeding to the next activity.

In task 11, “Reviewing Statistical Analysis Results,” 3 errors were identified. In subtask 11.1, “Reviewing Data Graphs,” no issues were observed. In subtask 11.2, “Reviewing Activity Data,” participants were unable to review the data because activity records were not saved, and there was inconsistency between the activity names on the playscreen and the statistical analysis screen, leading to further confusion. In subtask 11.3, “Deleting Data” participants clicked on the pie chart and searched for a delete button on the detailed graph screen.

### Survey Results

The survey assessing the usability of user interface showed that most participants rated usability as a score of 2 or lower, indicating ease of use on a 5-point Likert scale (1=very easy to 5=very difficult; Table 4).

**Table 4.** Survey results of usability of user interface (n=5). Usability of user interface showed that most participants rated usability as a score of 2 (easy) or lower (1=very easy; 5=very difficult).

Usability of UI^a^		Mean (SD)	
Reviewing user manual			
Identifying information		1.67 (SD 0.82)	
Comprehensibility		1.83 (SD 0.98)	
Checking the components			
Differentiating between components		1.50 (SD 0.55)	
Verifying connection and power		1.17 (SD 0.41)	
Launching the program			
Launching the software		1.00 (SD 0.00)	
Entering the password		1.00 (SD 0.00)	
Creating patient information			
Creating patient information		2.17 (SD 0.75)	
Consenting to the disclosure of personal information		1.67 (SD 1.03)	
Donning the product			
Donning the product		2.00 (SD 1.10)	
Instructions regarding the product		1.83 (SD 0.98)	
Running the upper rehabilitation content			
Engaging in the content		1.50 (SD 0.55)	
Explaining the contents		1.50 (SD 0.55)	
Performing the activity			
Pausing the content		1.67 (SD 1.03)	
Configuring detailed settings		2.17 (SD 1.33)	
Changing detailed settings		2.17 (SD 1.33)	
Ending the content		1.67 (SD 1.03)	
Updating patient information			
Updating patient information		1.33 (SD 0.82)	
Running contents after updating patient information			
Changing the patient		1.67 (SD 1.03)	
Doffing the product			
Doffing the product		1.50 (SD 0.84)	
Reviewing statistical analysis results			
Checking the graph		1.33 (SD 0.82)	
Checking data		1.33 (SD 0.82)	
Deleting data		1.50 (SD 1.22)	
Ending the program			
Ending the software		1.17 (SD 0.41)	

a UI: user interface.

## Discussion

### Principal Findings

The following recommendations were suggested from the results of the cognitive walkthrough to enhance usability and user satisfaction with the interface of the VR-based upper limb rehabilitation software. First, enhancements to the graphical user interface (GUI) are essential. A GUI facilitates more intuitive and efficient information presentation using graphics instead of text [24]. This study identified usability issues stemming from the lack of clarity in the “Add Patient” icon, underscoring the need for a more intuitive icon that clearly conveys its function. In addition, improvements in typography, such as optimizing font size and color contrast, are necessary to enhance the readability of activity-setting screens.

Furthermore, to streamline data entry, manual input for all patient information fields should be replaced with input controls such as pickers, which allow users to select from predefined options, or steppers, which enable users to increment or decrement numbers or values using “+” and “-” buttons. In addition, field navigation should be optimized by enabling users to switch between fields using the tab key, thereby facilitating user input. Finally, the functionality for controlling buttons within the software should be visibly displayed on the interface. When developing GUI-based medical software devices, the interface design should aim to integrate clear control mechanisms and simplify tasks, thereby minimizing users’ cognitive load and memory demands [25].

Second, integrating natural user interface (NUI) elements could further enhance usability. NUI refers to an interface that relies on gestures rather than traditional input devices, such as a mouse or keyboard, to control content [26]. While the VR-based upper limb rehabilitation software uses a gesture-based interface using an HMD and controllers, the system does not differentiate between left and right sides. This limitation can hinder therapists from properly administering treatments for patients with unilateral paralysis. To address this issue, the software should include features to either automatically detect the controller’s orientation or provide manual configuration options to distinguish the affected side. Furthermore, therapists reported difficulties in adjusting the perceived distance between the hand and objects displayed on their monitors, highlighting the need for improved distance control mechanisms in the VR environment. Therefore, a VR software interface should be designed to ensure ease of use for both therapists and patients.

Third, the user interface (UI) design requires improvements to enhance the interaction between the medical device and its users. In this study, we observed that therapists used inconsistent methods for adjusting settings and some failed to save their configurations before proceeding with the tasks. Although most medical device users are well-trained and experienced, errors can still occur when they rely heavily on their own previous knowledge. If the system can provide warning alerts when settings are unsaved or incorrect actions are detected, and provide confirmation prompts when actions are correct, unintended errors can be mitigated [27]. Therefore, warning alerts, confirmation popups, detailed instructions, and help guides within the software interface are recommended to prevent usage errors.

Formative evaluation is a type of usability test conducted during the medical device development phase to improve product design. In this study, we conducted a cognitive walkthrough and surveys with occupational therapists as participants to explore potential areas for improvement in the VR-based upper limb rehabilitation software.

Cognitive walkthrough is a usability test method used to systematically identify interactions between users and a system without previous knowledge of the system. It is particularly suitable for evaluating screen-based systems where users must actively navigate the interface [16]. Test tasks should be selected from the user’s perspective, and the sequence of potential actions should first be outlined, followed by a step-by-step analysis of continuous actions to assess whether appropriate actions are taken at the correct moments. Successful interaction is determined by whether the system feedback helps users achieve their intended goals [28].

In this study, we identified usability issues that need to be addressed in the UI of the VR-based upper limb rehabilitation software as a medical device. However, there are some limitations to note. Although both health care providers delivering the treatment and patients receiving it were considered users, actual patients were not included in the participant group. In addition, the therapists involved in the study did not represent a diverse range of clinical experience levels. Despite these limitations, the rehabilitation specialists who participated in this study provided valuable insights, including perspectives from the patients’ point of view. Future research should expand the scope of such work to include a broader range of users and conduct a more comprehensive analysis of the usability across various features and tasks.

### Conclusion

VR-based rehabilitation represents a rapidly emerging field in health care. In this study, we conducted a usability evaluation of a prototype VR-based upper limb rehabilitation software. By ensuring a user-centered interface and addressing the identified UI issues and areas for improvement, enhancing the usability of the software as a medical device will contribute to the development of a solution that fully meets the needs of its users. These efforts will not only prioritize user satisfaction and ease of use but also accelerate the commercialization of the VR-based upper limb rehabilitation software. Furthermore, improving user-device interaction will enhance the usability and satisfaction associated with the device, ultimately contributing to the development of a medical device that delivers error-free performance, as intended by the system.

## Abbreviations

GUI: graphical user interface
HMD: head-mounted display
NUI: natural user interface
UI: user interface
VR: virtual reality
